# Supplementary material for: Inertial delay of self-propelled particles
Source: Nat Commun. 2018 Dec 4;9:5156. doi: 10.1038/s41467-018-07596-x (PMC6279816; doi:10.1038/s41467-018-07596-x)
Supplement: Supplementary file 1 — Supplementary Information [file 41467_2018_7596_MOESM1_ESM.pdf]

Supplementary Information

Inertial delay of self-propelled particles

Scholz et al.

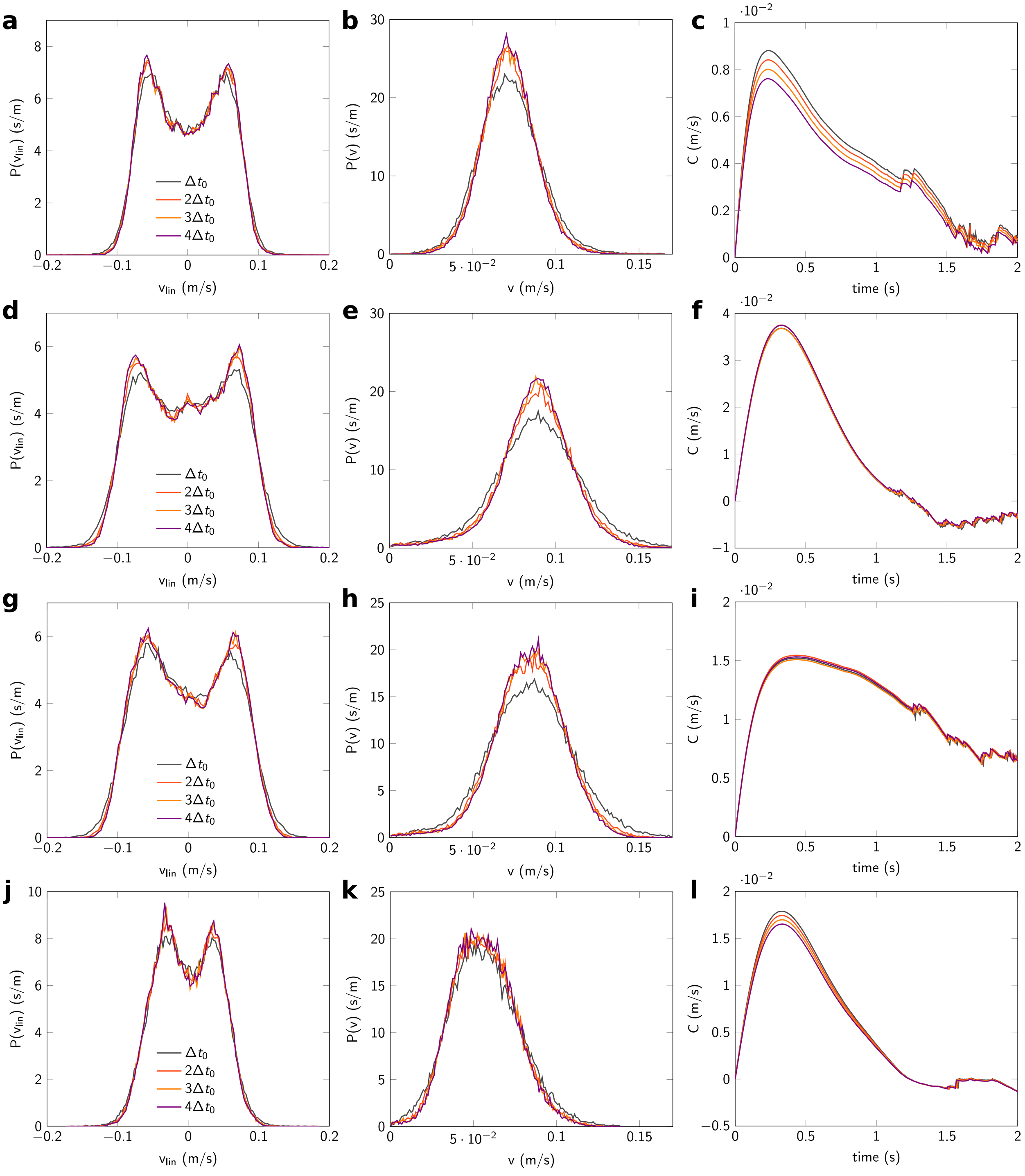

Supplementary Figure 1. **Linear and absolute velocity distributions and delay function.** (a-c) Generic particle, (d-e) carrier particle, (g-i) tug particle and (j-l) ring particle. The timescale Δt in the velocity definition is changed in the range $1, 2, 3, 4\,\Delta t_0$. Only small differences are observed in some curves, which lead only to minor changes in the fit parameters.

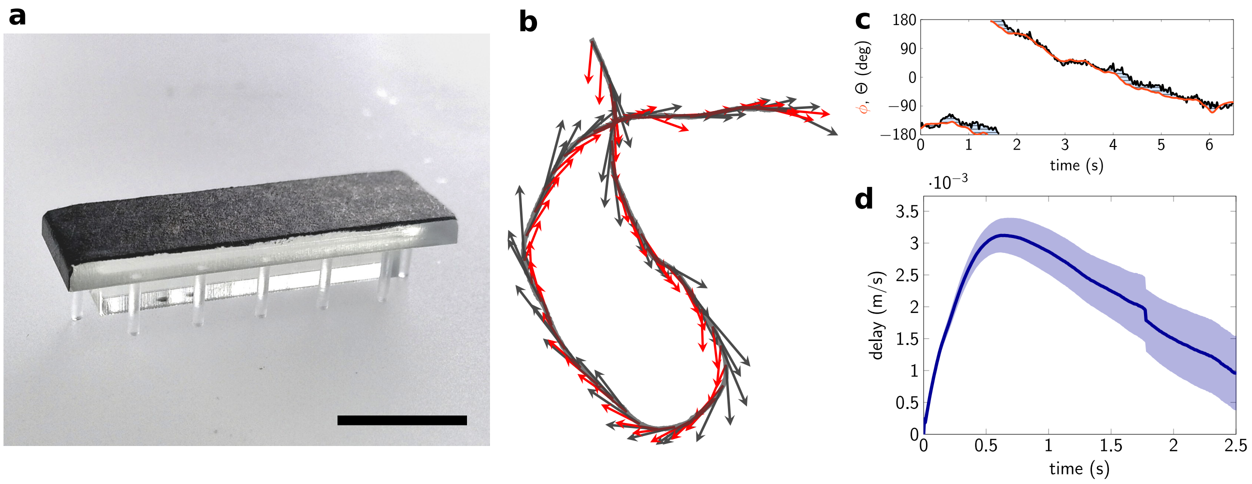

Supplementary Figure 2. **Inertial delay of a rod-like particle.** (a) 3D-printed rod-like vibrobot with aspect ratio three, printed from clear resin. Scale bar represents 10 mm. (b) Sample trajectory showing delay of velocity direction (black) and orientation angle (red). (c) Delay between velocity direction angle Θ (black) and orientation angle ϕ (red) demonstrates that Θ typically lags behind ϕ. (d) Delay function showing the characteristic shape.

Supplementary Table 1. Mass, moment of inertia and model parameters obtained from analytic solutions and fits to measurements in Fig. 3.

Particle	M [kg]	J [kg m^2]	V_p [m s^{-1}]	τ^{-1} [s^{-1}]	D [m^{2}s^{-1}]	D_r [rad^2 s^{-1}]	τ_r^{-1} [s^{-1}]	ω_p [rad s^{-1}]
Generic	0.76×10^{-3}	1.64×10^{-8}	0.071	9.3	3.56×10^{-5}	0.91	14.9	0.354
Carrier	4.07×10^{-3}	1.46×10^{-7}	0.0929	6.85	7.7×10^{-5}	2.7	5.1	0.714
Tug	1.57×10^{-3}	2.54×10^{-8}	0.087	3.0	2.2×10^{-4}	0.59	17.6	−0.614
Ring	0.33×10^{-3}	1.26×10^{-8}	0.057	5.0	8.4×10^{-5}	2.4	5.0	−0.19

Supplementary Table 2. Model parameters obtained from numerical fits to measurements in Fig. 3 and Fig. 4. Experimental uncertainty is specified in brackets in iso notation starting from the last respective digit.

Particle	V_p [m s^{-1}]	τ^{-1} [s^{-1}]	D [m^{2}s^{-1}]	D_r [rad^2 s^{-1}]	τ_r^{-1} [s^{-1}]	ω_p [rad s^{-1}]
Generic	0.0708(55)	12.45(211)	$2.48(105) \times 10^{-5}$	0.94(10)	15.20(454)	0.346(13)
Carrier	0.0954(76)	6.11(151)	$8.20(336) \times 10^{-5}$	2.73(35)	5.17(97)	0.734(28)
Tug	0.0846(72)	6.20(120)	$9.74(327) \times 10^{-5}$	0.60(7)	16.78(482)	−0.622(23)
Ring	0.0553(33)	7.15(107)	$5.90(117) \times 10^{-5}$	2.45(13)	4.98(122)	−0.181(4)

SUPPLEMENTARY METHODS

Analytic approximation of linear velocity distribution

One can obtain the time–dependent probability distribution through the corresponding Fokker–Planck equation which can be derived from Langevin equations as

$$
\begin{aligned}
-\frac{\partial}{\partial t}P(\mathfrak{R},t) &= \partial_X \dot{X} P(\mathfrak{R},t) + \partial_Y \dot{Y} P(\mathfrak{R},t) + \partial_\phi \dot{\phi} P(\mathfrak{R},t) \\
&+ \partial_{\dot{X}} \left(\frac{\xi}{m} V_{\mathrm{p}} \cos\phi - \frac{\xi}{m}\dot{X} - D\left(\frac{\xi}{m}\right)^2 \partial_{\dot{X}} \right) P(\mathfrak{R},t) \\
&+ \partial_{\dot{Y}} \left(\frac{\xi}{m} V_{\mathrm{p}} \sin\phi - \frac{\xi}{m}\dot{Y} - D\left(\frac{\xi}{m}\right)^2 \partial_{\dot{Y}} \right) P(\mathfrak{R},t) \\
&+ \partial_{\dot{\phi}} \left(\frac{\xi_{\mathrm{r}}}{J} \omega - \frac{\xi_{\mathrm{r}}}{J}\dot{\phi} - D_{\mathrm{r}}\left(\frac{\xi_{\mathrm{r}}}{J}\right)^2 \partial_{\dot{\phi}} \right) P(\mathfrak{R},t)\,, \qquad (1)
\end{aligned}
$$

with $\mathfrak{R} = \begin{pmatrix} X & Y & \phi & \dot{X} & \dot{Y} & \dot{\phi} \end{pmatrix}$. The stationary distribution is obtained by setting the r.h.s of Supplementary Eq. (1) to zero. Since we are interested in the stationary linear velocity distribution along (for instance) X direction, we integrate the other variables such that the Fokker Planck equation reduces to

$$
\int_{-\infty}^{\infty} d\phi\, \partial_{\dot{X}} \left(\frac{\xi}{m} V_{\mathrm{p}} \cos\phi - \frac{\xi}{m}\dot{X} - D\left(\frac{\xi}{m}\right)^2 \partial_{\dot{X}} \right) P\left(\dot{X},\phi\right) = 0\,. \qquad (2)
$$

Because the stationary distribution of the angle ϕ is uniform, based on Supplementary Eq. (2), one can anticipate the linear velocity distribution to be in the following form

$$
P\left(\dot{X}\right) = \frac{1}{\sqrt{2\pi q}} \int_{-\pi}^{\pi} d\phi\, \frac{1}{2\pi} \exp\left(-\frac{\left(\dot{X} - W\cos\phi\right)^2}{2q} \right)\,. \qquad (3)
$$

In analogy to [1], via computing the respective second and forth moments and by using the ansatz of Supplementary Eq. (3),

$$
\begin{aligned}
\left\langle \dot{X}^2 \right\rangle &= q + \frac{1}{2}W^2\,, \\
\left\langle \dot{X}^4 \right\rangle &= 3q^2 + 3qW^2 + \frac{3}{8}W^4\,, \qquad (4)
\end{aligned}
$$

it is deduced that under the condition

$$
\mathcal{E}_c = \frac{|\mathfrak{W}^4 - \mathfrak{w}_{\mathrm{dev}}|}{\mathfrak{W}^4} \ll 1\,, \qquad (5)
$$

W and q can be evaluated as

$$W = \mathfrak{W} + \frac{\mathfrak{W}^4 - \mathfrak{w}_{\rm dev}}{4\mathfrak{W}^3}\,,$$
$$q = D\frac{\xi}{m} - \frac{\mathfrak{W}^4 - \mathfrak{w}_{\rm dev}}{4\mathfrak{W}^2}\,, \tag{6}$$

where

$$\mathfrak{W} = V_{\rm p}\sqrt{\mathfrak{f}(\mathfrak{D}_0, \mathfrak{D}_1\,, \mathfrak{D}_2)} \tag{7}$$

and

$$\begin{aligned}\mathfrak{w}_{\rm dev} = \mathfrak{D}_2^2 V_{\rm p}^4 e^{2\mathfrak{D}_0}\mathfrak{D}_0^{-2(2\mathfrak{D}_2+\mathfrak{D}_0)}\,\mathrm{Re}\Bigg[&\gamma(3\mathfrak{D}_2 + \mathfrak{D}_0 - i\mathfrak{D}_1, \mathfrak{D}_0)\\ \times \Bigg[&\gamma(\mathfrak{D}_2 + \mathfrak{D}_0 + i\mathfrak{D}_1, \mathfrak{D}_0) + \mathfrak{D}_0^{2i\mathfrak{D}_1}\gamma(\mathfrak{D}_2 + \mathfrak{D}_0 - i\mathfrak{D}_1, \mathfrak{D}_0)\\ \times \Bigg(&1 + 2\mathfrak{D}_2 e^{4\mathfrak{D}_0}(4\mathfrak{D}_0)^{-2(\mathfrak{D}_2+2\mathfrak{D}_0-i\mathfrak{D}_1)}\\ \times\, &\gamma(2\mathfrak{D}_2 + 4\mathfrak{D}_0 - 2i\mathfrak{D}_1, 4\mathfrak{D}_0)\Bigg)\Bigg]\Bigg]\,,\end{aligned} \tag{8}$$

where $\mathfrak{f}$ is given by Eq. (7) in the main manuscript.

SUPPLEMENTARY REFERENCES

[1] U. Basu, S. N. Majumdar, A. Rosso, and G. Schehr, Preprint at <https://arxiv.org/abs/1804.09027> (2018).
