# Supplementary material for: Inertial delay of self-propelled particles
Source: Nat Commun. 2018 Dec 4;9:5156. doi: 10.1038/s41467-018-07596-x (PMC6279816; doi:10.1038/s41467-018-07596-x)
Supplement: Supplementary file 3 — Description of Additional Supplementary Files [file 41467_2018_7596_MOESM3_ESM.pdf]

Description of supplementary files:

Supplementary Movie 1

Illustration of the experimental setup and the motion mechanism of the particle.

Supplementary Movie 2

Recording of three representative measurements of the motion of a carrier particle.

Supplementary Movie 3

Measurement of particle orientation and velocity, demonstrating the systematic shift between the two quantities.
